# Metabolic dysfunction-associated steatotic liver disease increases the risk of acute kidney injury in septic shock: A United States population-based study

**DOI:** 10.1097/MD.0000000000048142

**Published:** 2026-03-27

**Authors:** Guy Loic Nguefang Tchoukeu, Sarpong Boateng, Yussif Issaka, Elvis Eze, Yazan A. Al-Ajlouni, Prince A. Ameyaw, Edgar Luna Landa, Anjul Verma, Avneet Kaur, Joel Gabin Konlack Mekontso, Basile Njei

**Affiliations:** a Department of Internal Medicine, Texas Tech University Health Sciences Center, Odessa, TX; b Department of Internal Medicine, Yale New Haven Health, Bridgeport Hospital, New Heaven, CT; c Department of Internal Medicine, Yale School of Medicine, Yale New Haven Hospital, New Heaven, CT; d Department of Rehabilitation, Montefiore Medical Center, Bronx, NY; e Department of Internal Medicine, Upstate University Hospital, Syracuse, NY; f Department of Internal Medicine, South Brooklyn Health, Brooklyn, NY.

**Keywords:** critical care outcomes, liver disease, metabolic dysfunction-associated steatotic liver disease (MASLD), sepsis, septic shock

## Abstract

Metabolic dysfunction-associated steatotic liver disease (MASLD) is associated with systemic inflammation and potentially influences the outcomes of critical illness. However, its impact on septic shock remains poorly defined. We aimed to evaluate the association between MASLD score and outcomes in patients with septic shock. We conducted a retrospective cohort study using the National Inpatient Sample (2016–2020) and included adults (aged ≥ 18 years) with septic shock. Patients were stratified according to the MASLD status, and those with acute liver diseases were excluded. A 1:1 propensity score matching was performed based on the demographics, comorbidities, and hospital characteristics. Outcomes included mortality, acute kidney injury (AKI), pulmonary embolism, cardiac arrest, acute respiratory distress syndrome, and transfusion requirements. Multivariable logistic regression was performed to assess the association between the MASLD score and outcomes. Among the 17,382 patients with septic shock, 8691 had MASLD. The incidence of AKI was higher in the MASLD compared than in the non-MASLD (69.9% vs 68.4%, *P* = .036). In contrast, MASLD was associated with lower mortality (27.8% vs 33.0%; *P* < .0001), cardiac arrest (5.3% vs 8.2%; *P* < .0001), acute respiratory distress syndrome (32.3% vs 36.4%; *P* < .0001), pulmonary embolism (2.2% vs 4.2%; *P* < .0001), and transfusion requirements (19.6% vs 21.3%, *P* = .005). MASLD had higher odds of AKI (adjusted odds ratio 1.071; 95% confidence interval, 1.005–1.143; *P* = .036), particularly in black patients (adjusted odds ratio 1.327; 95% confidence interval, 1.072–1.642; *P* = .0092). MASLD was associated with higher odds of AKI but paradoxically lower odds of other adverse outcomes. Further research is required to elucidate the mechanisms linking MASLD to septic shock outcomes.

## 1. Introduction

Metabolic dysfunction-associated steatotic liver disease (MASLD), formerly known as nonalcoholic fatty liver disease, is the most prevalent chronic liver disease globally, affecting over 30% of adults, with its prevalence rising alongside obesity and metabolic syndrome. MASLD encompasses a spectrum of conditions ranging from simple steatosis to metabolic dysfunction-associated steatohepatitis, which can progress to cirrhosis, liver failure, and hepatocellular carcinoma.^[1-7]^ Beyond liver-specific complications, MASLD is recognized as a systemic disorder precipitating cardiovascular disease, chronic kidney disease, and heightened overall morbidity and mortality.^[1,6,8,9]^

Sepsis remains a major public health concern and is the most prevalent diagnosis among intensive care unit (ICU) admissions.^[10-12]^ In the United States (US), sepsis accounts for approximately 13% of all hospitalizations.^[12]^ Septic shock, the most severe form of sepsis, is associated with high mortality rates, estimated at 34.2% in the US and 36.7% in Europe.^[12-14]^ Despite advances in critical care, septic shock continues to impose a substantial clinical and economic burden due to prolonged ICU stays, multi-organ failure, and high treatment cost.^[10,11]^

Individuals with MASLD may exhibit increased susceptibility to infections and heightened inflammatory responses.^[1,15,16]^ Although prior studies have examined the relationship between MASLD and sepsis most were limited by retrospective or single-center designs and did not specifically address septic shock.^[1,15-17]^ Consequently, the impact of MASLD on outcomes in patients with septic shock remains poorly understood. Whether the metabolic and inflammatory milieu associated with MASLD exacerbates or modifies clinical trajectories in septic shock has yet to be clearly established.

To address this knowledge gap, we conducted a large, nationally representative analysis using the National Inpatient Sample (NIS) from 2016 to 2020 to evaluate the impact of MASLD on in-hospital outcomes among patients with septic shock.

## 2. Materials and methods

### 2.1. Data source

This retrospective cohort study utilized data from the NIS database from 2016 to 2020, developed by the Healthcare Cost and Utilization Project sponsored by the Agency for Healthcare Research and Quality. The NIS is the largest publicly available all-payer inpatient care database in the United States, capturing approximately 20% of all inpatient discharges from US community hospitals, excluding rehabilitation and long-term acute-care hospitals. This dataset allows for the generation of nationally representative estimates of hospitalizations across the Unitd States.^[18]^

### 2.2. Study population

We identified adult patients (≥18 years) hospitalized with a primary diagnosis of septic shock, defined using International Classification of Diseases, Tenth Revision, Clinical Modification (ICD-10-CM) codes. Hospitalizations were excluded if patients had documented acute liver diseases or secondary causes of hepatic steatosis, including acute viral hepatitis, alcoholic liver disease, autoimmune hepatitis, biliary liver disease, or other non-metabolic etiologies of liver disease. Patients meeting inclusion criteria were stratified according to the presence or absence of MASLD.

### 2.3. Definition of MASLD

MASLD was defined using ICD-10-CM diagnosis codes consistent with contemporary nomenclature and prior epidemiologic studies. ICD-10-based definitions are widely used and validated for large population-based studies using administrative datasets such as the NIS.^[19]^ Codes corresponding to hepatic steatosis in the context of metabolic dysfunction were used, while secondary causes of steatosis were explicitly excluded. The full list of ICD-10-CM codes used to define MASLD is provided in Table S1, Supplemental Digital Content, https://links.lww.com/MD/R576.

### 2.4. Variables and covariates

The demographic variables included age, sex, race/ethnicity, primary insurance payer, and hospital-level characteristics. To minimize confounding, 1:1 propensity score matching was performed between MASLD and non-MASLD patients based on key demographics, comorbidities, and hospital characteristics using a nearest-neighbor algorithm without replacement and a caliper width of 0.2 standard deviations of the logit of the propensity score.

### 2.5. Study outcomes

The primary outcome was inpatient mortality. The secondary outcomes included acute kidney injury (AKI), cardiac arrest, acute respiratory distress syndrome (ARDS), pulmonary embolism, and transfusion requirements.

### 2.6. Statistical analysis

Analyses were conducted using a complete-case approach without imputation for missing data, consistent with standard practices for analyses of the NIS. Descriptive statistics were used to compare the demographic and clinical characteristics of the matched MASLD and non-MASLD cohorts. Continuous variables were presented as means with standard deviations and compared using the Kruskal–Wallis test. Categorical variables were expressed as frequencies and percentages and were compared using the chi-square test. Multivariable logistic regression models were used to assess the independent association between the MASLD score and each outcome. Adjusted odds ratios (aOR) with corresponding 95% confidence intervals (CIs) and *P*-values are reported. All statistical analyses were conducted using SAS version 9.4. Statistical significance was set at a 2-tailed *P*-value < .05.

### 2.7. Sensitivity analyses

To assess the robustness of our findings, we performed prespecified sensitivity analyses. First, we excluded patients with a documented history of chronic kidney disease prior to hospitalization to minimize potential confounding related to baseline renal dysfunction. Second, we repeated the propensity score matching using a more stringent caliper width of 0.1 standard deviations of the logit of the propensity score. Associations between MASLD and clinical outcomes were re-estimated using these alternative specifications.

### 2.8. Ethical considerations

This study used data from the NIS, a publicly available, de-identified database. As such, this study was exempt from Institutional Review Board review, and formal ethical approval was not required. Informed consent was not obtained because the NIS does not contain identifiable patient information. The study followed the Strengthening the Reporting of Observational Studies in Epidemiology guidelines.

### 2.9. Data availability statement

The data supporting these findings are publicly available from the Agency for Healthcare Research and Quality. The NIS can be accessed at https://hcup-us.ahrq.gov/nisoverview.jsp following the Healthcare Cost and Utilization Project data use agreement and purchase procedure. The authors have no special access privileges.

## 3. Results

### 3.1. Baseline characteristics

**Figure 1.**
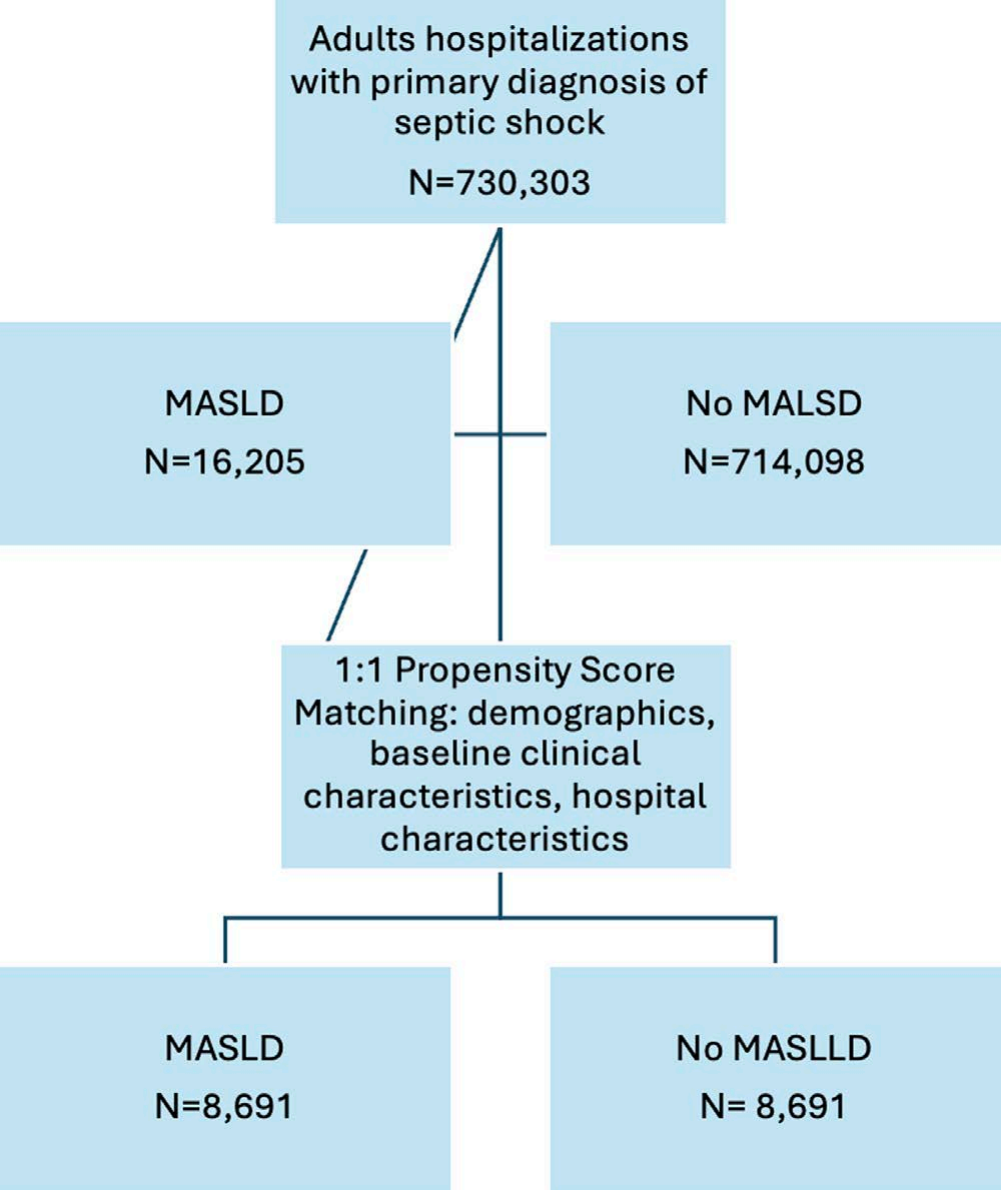
Flowchart for selection of study cohort. Flow diagram illustrating the identification of adult hospitalizations with a primary diagnosis of septic shock (N = 730,303). Patients were stratified based on the presence or absence of metabolic dysfunction-associated steatotic liver disease (MASLD). A total of 16,205 hospitalizations with MASLD and 714,098 without MASLD were identified. After 1:1 propensity score matching adjusting for demographics, baseline clinical characteristics, and hospital characteristics, 8691 matched hospitalizations remained in each cohort for the final analysis. MASLD = metabolic dysfunction-associated steatotic liver disease.

A total of 17,382 adult patients hospitalized with septic shock were included in the final matched cohort, with 8691 patients in each group based on the presence or absence of MASLD (Fig. 1). Propensity score matching resulted in well-balanced demographic and clinical characteristics between the groups. The mean age was comparable between the MASLD and non-MASLD cohorts (59.9 vs 59.8 years, *P* = .196). The racial distribution was similar between groups, with White patients comprising the majority (69.7% vs 69.8%, *P* = .717), followed by Hispanic, Black, and Asian/Pacific Islander populations, indicating adequate balance across racial and ethnic subgroups for subsequent stratified analyses (Table 1).

**Table 1:** Baseline demographic and clinical characteristics after propensity score matching.

Characteristics	Without MASLD	With MASLD	Total	*P*-value
Age	(N = 8691)	(N = 8691)	(N = 17382)	.1962*
Mean (SD)	59.8 (15.57)	59.9 (13.70)	59.8 (14.67)	
Median	62.0	61.0	62.0	
Range	18.0; 90.0	18.0; 90.0	18.0; 90.0	
Total charges	(N = 8613)	(N = 8612)	(N = 17225)	<.0001*
Mean (SD)	2,22,975.3 (3,52,158.67)	1,72,936.1 (2,39,854.18)	1,97,957.2 (3,02,316.97)	
Median	1,24,351.0	101999.0	1,11,731.0	
Range	120.0; 91,88,020.0	1026.0; 53,07,472.0	120.0; 91,88,020.0	
Length of stay	(N = 8691)	(N = 8689)	(N = 17380)	<.0001*
Mean (SD)	13.8 (15.96)	11.6 (12.28)	12.7 (14.29)	
Median	9.0	8.0	9.0	
Range	0.0, 292.0	0.0, 198.0	0.0, 292.0	
Primary payer, n (%)	(N = 8691)	(N = 8691)	(N = 17382)	.9988†
Private insurance	4346 (50.0%)	4324 (49.8%)	8670 (49.9%)	
Medicaid	1515 (17.4%)	1510 (17.4%)	3025 (17.4%)	
Medicare	2220 (25.5%)	2247 (25.9%)	4467 (25.7%)	
Other payment source	362 (4.2%)	362 (4.2%)	724 (4.2%)	
Self-pay	33 (0.4%)	33 (0.4%)	66 (0.4%)	
No charge	215 (2.5%)	215 (2.5%)	430 (2.5%)	
Race n (%)	(N = 8691)	(N = 8691)	(N = 17382)	.7166†
White	6062 (69.8%)	6056 (69.7%)	12,118 (69.7%)	
Black	767 (8.8%)	791 (9.1%)	1558 (9.0%)	
Hispanic	1234 (14.2%)	1196 (13.8%)	2430 (14.0%)	
Asian/Pacific Islander	244 (2.8%)	266 (3.1%)	510 (2.9%)	
Native American	113 (1.3%)	125 (1.4%)	238 (1.4%)	
Other	271 (3.1%)	257 (3.0%)	528 (3.0%)	
Region of hospital, n (%)	(N = 8691)	(N = 8691)	(N = 17382)	.1388†
Northeast	963 (11.1%)	1042 (12.0%)	2005 (11.5%)	
Midwest	2143 (24.7%)	2061 (23.7%)	4204 (24.2%)	
South	3597 (41.4%)	3554 (40.9%)	7151 (41.1%)	
West	1988 (22.9%)	2034 (23.4%)	4022 (23.1%)	
Elixhauser comorbidity	(N = 8691)	(N = 8691)	(N = 17382)	.0114†
Mean (SD)	6.4 (2.26)	6.4 (2.12)	6.4 (2.19)	
Median	6.0	6.0	6.0	
Range	0.0, 15.0	0.0, 15.0	0.0, 15.0	

Continuous variables are presented using mean (standard deviation), median, and range. Categorical variables are presented as frequencies and percentages. *P*-values were calculated using the Kruskal–Wallis test for continuous variables and the Chi-square test for categorical variables.

SD = standard deviation, MASLD = metabolic dysfunction-associated steatotic liver disease.

* Kruskal–Wallis *P*-value.

† Chi-square *P*-value.

### 3.2. Clinical outcomes

Patients with MASLD demonstrated higher unadjusted rates of in-hospital mortality compared to those without MASLD (33.0% vs 27.8%; *P* < .0001). The incidence of AKI was also higher in the MASLD group (69.9% vs 68.4%, *P* = .036). However, patients with MASLD had lower incidence rates of cardiac arrest (5.3% vs 8.2%, *P* < .0001), ARDS or mechanical ventilation (32.3% vs 36.4%, *P* < .0001), pulmonary embolism (2.2% vs 4.2%, *P* < .0001), and transfusion requirement (19.6% vs 21.3%, *P* = .005; Table 2).

**Table 2:** Unadjusted clinical outcomes of patients with and without MASLD.

Outcomes	Without MASLD (N = 8691)	With MASLD (N = 8691)	Total (N = 17382)	*P*-value
Cardiac arrest, n (%)	715 (8.2%)	461 (5.3%)	1176 (6.8%)	<.0001*
AKI, n (%)	5946 (68.4%)	6074 (69.9%)	12,020 (69.2%)	.0355*
Pulmonary embolism, n (%)	361 (4.2%)	192 (2.2%)	553 (3.2%)	<.0001*
ARDS, n (%)	3165 (36.4%)	2810 (32.3%)	5975 (34.4%)	<.0001*
Transfusion, n (%)	1848 (21.3%)	1700 (19.6%)	3548 (20.4%)	.0054*
Death, n (%)	2411 (27.8%)	2869 (33.0%)	5280 (30.4%)	<.0001*

Outcomes are presented as number (percentage). *P*-values were calculated using the chi-square test. A 2-sided *P*-value < .05 was considered statistically significant.

AKI = acute kidney injury, ARDS = acute respiratory distress syndrome, MASLD = metabolic dysfunction-associated steatotic liver disease.

* Chi-square *P*-value.

In multivariable logistic regression analysis, MASLD was independently associated with higher odds of in-hospital mortality (aOR 1.28; 95% CI, 1.20–1.37; *P* < .0001) and AKI (aOR 1.07; 95% CI, 1.00–1.14; *P* = .036). In sensitivity analyses excluding patients with preexisting chronic kidney disease, MASLD remained significantly associated with higher odds of death and AKI, with estimates similar in magnitude to those observed in the primary analysis. Likewise, repeating the propensity score matching using a narrower caliper width of 0.1 yielded consistent results for all primary and secondary outcomes. (Tables S2 and S3, Supplemental Digital Content, https://links.lww.com/MD/R576).

MASLD was associated with significantly lower odds of cardiac arrest (aOR 0.62; 95% CI, 0.55–0.70; *P* < .0001), ARDS (aOR 0.83; 95% CI, 0.78–0.88; *P* < .0001), pulmonary embolism (aOR 0.52; 95% CI, 0.43–0.62; *P* < .0001) and transfusion (aOR 0.90; 95% CI, 0.83–0.97; *P* < .0001; Table 3).

**Table 3:** Adjusted odds ratios for outcomes among patients with MASLD.

Outcome	aOR	Lower CL	Upper CL	*P*-value
Cardiac arrest	0.62	0.55	0.70	<.0001
AKI	1.07	1.00	1.14	0.036
Pulmonary embolism	0.52	0.43	0.62	<.0001
ARDS	0.83	0.78	0.88	<.0001
Transfusion	0.90	0.83	0.97	.0051
Death	1.28	1.20	1.37	<.0001

Adjusted odds ratios are presented with 95% confidence limits. *P*-values were derived from multivariable logistic regression models adjusted for baseline demographic and clinical characteristics. A 2-sided *P*-value < .05 was considered statistically significant.

AKI = acute kidney injury, aOR = adjusted odds ratio, ARDS = acute respiratory distress syndrome, CL = confidence limits, MASLD = metabolic dysfunction-associated steatotic liver disease.

In race-stratified analyses, MASLD was associated with significantly higher odds of AKI among Black patients (aOR 1.33; 95% CI, 1.07–1.64; *P* = .0092), whereas no statistically significant association was observed among other races (Table 4).

**Table 4:** Adjusted odds ratios for clinical outcomes by race.

Race	Outcome	aOR	Lower CL	Upper CL	*P*-value
White	Cardiac Arrest	0.61	0.53	0.72	<.0001
Black	Cardiac Arrest	0.81	0.60	1.10	.187
Hispanic	Cardiac Arrest	0.46	0.33	0.64	<.0001
Asian/Pacific Islander	Cardia c Arrest	0.74	0.36	1.54	.422
Native American	Cardiac Arrest	0.90	0.25	3.19	.870
Other	Cardiac Arrest	0.61	0.31	1.18	.144
White	AKI	1.05	0.97	1.13	.259
Black	AKI	1.33	1.07	1.64	.009
Hispanic	AKI	1.07	0.90	1.26	.451
Asian/Pacific Islander	AKI	1.31	0.92	1.88	.134
Native American	AKI	0.77	0.46	1.30	.335
Other	AKI	1.00	0.68	1.46	.994
White	ARDS	0.85	0.79	0.92	<.0001
Black	ARDS	0.93	0.76	1.14	.491
Hispanic	ARDS	0.65	0.55	0.78	<.0001
Asian/Pacific Islander	ARDS	1.05	0.72	1.52	.790
Native American	ARDS	0.92	0.54	1.58	.770
Other	ARDS	0.73	0.51	1.04	.081
White	Transfusion	0.92	0.83	1.00	.061
Black	Transfusion	1.02	0.82	1.27	.859
Hispanic	Transfusion	0.85	0.71	1.03	.096
Asian/Pacific Islander	Transfusion	0.65	0.43	0.97	.036
Native American	Transfusion	1.36	0.71	2.61	.351
Other	Transfusion	0.65	0.45	0.95	.028
White	Pulmonary embolism	0.47	0.38	0.58	<.0001
Black	Pulmonary embolism	1.04	0.61	1.78	.881
Hispanic	Pulmonary embolism	0.58	0.35	0.95	.030
Asian/Pacific Islander	Pulmonary embolism	0.22	0.05	1.06	.059
Native American	Pulmonary embolism	<0.001	<0.001	>999.99	.958
Other	Pulmonary embolism	0.52	0.19	1.40	.192
White	Death	0.80	0.74	0.87	<.0001
Black	Death	0.81	0.65	0.99	.045
Hispanic	Death	0.68	0.57	0.81	<.0001
Asian/Pacific Islander	Death	0.71	0.47	1.06	.091
Native American	Death	0.60	0.35	1.05	.075
Other	Death	0.73	0.50	1.06	.097

Adjusted odds ratios are presented with 95% confidence limits. *P*-values were derived from multivariable logistic regression models adjusted for baseline demographic and clinical characteristics. Race-stratified analyses were performed using the non-MASLD group as the reference within each racial category. A 2-sided *P*-value < .05 was considered statistically significant.

AKI = acute kidney injury, aOR = adjusted odds ratio, ARDS = acute respiratory distress syndrome, CL = confidence limits, MASLD = metabolic dysfunction-associated steatotic liver disease.

## 4. Discussion

In this large, propensity score–matched analysis of adults hospitalized with septic shock, MASLD was independently associated with higher mortality and an increased risk of AKI, with the strongest association observed among Black patients. In contrast, MASLD was associated with lower odds of cardiac arrest, ARDS, pulmonary embolism, and transfusion. While the elevated risks of mortality and AKI are consistent with the hypothesis that MASLD exacerbates organ vulnerability and systemic inflammation during critical illness, the inverse associations observed for other clinical outcomes warrant careful interpretation.

Our findings regarding AKI align with previous nationwide sepsis analyses and ICU-based studies, which have consistently shown that MASLD increases susceptibility to renal injury.^[1]^ The proposed mechanisms include chronic inflammation, endothelial dysfunction, insulin resistance, and heightened release of pro-inflammatory cytokines.^[20-22]^ Unlike earlier investigations that examined sepsis broadly, our study isolates septic shock as a distinct clinical entity, thereby uncovering an additional layer of complexity and risk associated with MASLD in this severe subset of critical illness.

Previous studies have shown that MASLD is associated with structural alterations in the liver that impair microbial clearance and disrupt innate immunity.^[1]^ Hepatic lipid accumulation may further promote neutrophil activation and amplify the inflammatory cascade in septic shock.^[23,24]^ This baseline pro-inflammatory milieu may predispose patients with MASLD to exaggerated cytokine release during septic shock, thereby exacerbating organ dysfunction and increasing mortality risk.^[25]^

The protective associations observed between MASLD and lower odds of cardiac arrest, pulmonary embolism, transfusion requirement, and ARDS have not been reported in prior studies examining MASLD and sepsis.^[1,20]^ Potential explanations include residual confounding, differences in treatment intensity – such as withdrawal of care or do not resuscitate status – and competing risks, whereby earlier mortality from other causes reduces the likelihood of these complications being captured. Given the complexity of these interactions and the inherent limitations of administrative datasets, including variability in coding practices across hospitals, prospective investigations are needed to clarify these unexpected associations.

The results of our study underscore the importance of recognizing MASLD as a potential risk modifier for the management of septic shock. Given its association with increased mortality and AKI, MASLD status should be considered when risk-stratifying patients in the ICU. Integrating MASLD into sepsis prognostic models may improve the early identification of high-risk patients and facilitate timely and tailored interventions.

Clinically, our findings support heightened mortality and surveillance for renal injury in MASLD patients with septic shock, including proactive hemodynamic optimization, avoidance of nephrotoxic exposures, and early nephrology consultation when appropriate. At the population level, the rising prevalence of MASLD suggests that its impact on ICU resource utilization and clinical outcomes will continue to expand. These observations reinforce the broader public health imperative to prioritize MASLD prevention and management as a central component of critical care strategies.

### 4.1. Strengths, bias, and confounding

A major strength of this study is the use of a large, nationally representative cohort derived from the National Inpatient Sample, which enhances the generalizability of our findings to the US population. The application of 1:1 propensity score matching enabled robust balancing of key demographic, clinical, and hospital-level characteristics between patients with and without MASLD, thereby reducing confounding by indication and strengthening causal inference.

Several strategies were implemented to further minimize bias. Sensitivity analyses excluding patients with preexisting chronic kidney disease and using a narrower caliper width demonstrated the robustness of the findings. As with all administrative databases, misclassification bias related to ICD-10 coding is possible; however, we used established code definitions and explicitly excluded secondary causes of liver disease to improve diagnostic specificity. Selection bias was mitigated by including all eligible hospitalizations meeting prespecified criteria within a nationally representative sample. Together, these methodological strengths and bias-mitigation strategies support the validity and applicability of the study results.

### 4.2. Limitations

Several limitations should be acknowledged when interpreting our findings. First, the retrospective design and reliance on administrative ICD-10 coding to define both MASLD and clinical outcomes introduce the possibility of misclassification bias. Although such definitions are widely used in large database studies, they may not fully capture disease severity or diagnostic nuance. Second, the NIS lacks granular clinical data, including laboratory values, illness severity scores (e.g., APACHE II, Sequential Organ Failure Assessment), hemodynamic parameters, and the timing of key interventions, which limits our ability to assess physiologic mechanisms and temporal relationships. Third, we were unable to stratify patients by MASLD stage, degree of fibrosis, or presence of steatohepatitis, all of which may differentially influence outcomes in critical illness. Fourth, although we employed propensity score matching, multivariable adjustment, and sensitivity analyses, residual confounding from unmeasured variables remains possible, as in all observational studies. Fifth, our analyses were conducted using a complete-case approach, and missing data could have introduced selection bias, although missingness was low and balanced between groups. Finally, variability in coding practices across institutions may have influenced the ascertainment of secondary diagnoses such as ARDS and cardiac arrest.

### 4.3. Future research

Future studies should focus on prospective studies that incorporate detailed clinical and biochemical data to characterize the relationship between MASLD and septic shock outcomes. Investigations are needed to elucidate the biological basis of the paradoxical associations observed for cardiac arrest, pulmonary embolism, transfusion requirements, and ARDS. Studies stratifying patients by MASLD stage, degree of fibrosis, and the presence of comorbidities could clarify whether disease severity modifies the risk profile of septic shock.

## 5. Conclusion

In conclusion, this analysis highlights MASLD as an important modifier of outcomes in septic shock, characterized by an increased risk of mortality and AKI. These findings carry significant clinical implications, emphasizing the need for heightened surveillance and the early implementation of kidney-protective strategies in septic shock patients with MASLD. From a public health perspective, MASLD imposes a substantial and growing burden on healthcare systems, underscoring the importance of integrating metabolic liver disease into critical care risk-stratification frameworks. Future research should prioritize prospective validation, mechanistic studies to further elucidate the pathways underlying these associations, and evaluation of targeted interventions aimed at improving outcomes in this expanding patient population.

## Author contributions

**Conceptualization:** Guy Loic Nguefang Tchoukeu, Sarpong Boateng, Elvis Eze, Yazan A. Al-Ajlouni, Prince A. Ameyaw, Edgar Luna Landa, Anjul Verma, Avneet Kaur, Joel Gabin Konlack Mekontso, Basile Njei.

**Data curation:** Guy Loic Nguefang Tchoukeu, Sarpong Boateng, Yazan A. Al-Ajlouni, Edgar Luna Landa, Anjul Verma, Avneet Kaur.

**Formal analysis:** Guy Loic Nguefang Tchoukeu, Sarpong Boateng, Elvis Eze, Prince A. Ameyaw, Edgar Luna Landa, Joel Gabin Konlack Mekontso.

**Funding acquisition:** Guy Loic Nguefang Tchoukeu.

**Investigation:** Guy Loic Nguefang Tchoukeu, Sarpong Boateng, Yussif Issaka, Yazan A. Al-Ajlouni, Edgar Luna Landa, Avneet Kaur.

**Methodology:** Guy Loic Nguefang Tchoukeu, Sarpong Boateng, Yussif Issaka, Elvis Eze, Prince A. Ameyaw, Edgar Luna Landa, Anjul Verma, Avneet Kaur, Joel Gabin Konlack Mekontso, Basile Njei.

**Project administration:** Guy Loic Nguefang Tchoukeu, Sarpong Boateng, Avneet Kaur, Joel Gabin Konlack Mekontso, Basile Njei.

**Resources:** Guy Loic Nguefang Tchoukeu, Yussif Issaka, Joel Gabin Konlack Mekontso, Basile Njei.

**Software:** Guy Loic Nguefang Tchoukeu, Sarpong Boateng, Elvis Eze, Yazan A. Al-Ajlouni, Prince A. Ameyaw, Basile Njei.

**Supervision:** Guy Loic Nguefang Tchoukeu, Yussif Issaka, Elvis Eze, Basile Njei.

**Validation:** Guy Loic Nguefang Tchoukeu, Basile Njei.

**Visualization:** Guy Loic Nguefang Tchoukeu, Elvis Eze, Basile Njei.

**Writing – original draft:** Guy Loic Nguefang Tchoukeu, Sarpong Boateng, Yussif Issaka, Anjul Verma, Basile Njei.

**Writing – review & editing:** Guy Loic Nguefang Tchoukeu, Basile Njei.

## Supplementary Material


