# Supplementary material for: Metabolic dysfunction-associated steatotic liver disease increases the risk of acute kidney injury in septic shock: A United States population-based study
Source: Medicine (Baltimore). 2026 Mar 27;105(13):e48142. doi: 10.1097/MD.0000000000048142 (PMC13034902; doi:10.1097/MD.0000000000048142)
Supplement: Supplementary file 1 [file medi-105-e48142-s001.docx]

Supplementary Tables

**Table S1:** ICD-10-CM codes used for inclusion and exclusion criteria in the study.

| **Category** | **ICD-10-CM Code(s)** | **Description** |
| --- | --- | --- |
| **Inclusion Criteria** | **Septic Shock** | **R65.21** – Severe sepsis with septic shock |
| **Exposure Variable** | **MASLD** | K76.0 – Fatty (change of) liver, not elsewhere classified (aligned with MASLD definition) |
| **Exclusion Criteria** |  |  |
| Cirrhosis & Advanced Fibrosis | K74.00 | Unspecified hepatic fibrosis |
|  | K74.01 | Early hepatic fibrosis |
|  | K74.02 | Advanced hepatic fibrosis |
|  | K74.1 | Hepatic sclerosis |
|  | K74.2 | Hepatic fibrosis with hepatic sclerosis |
|  | K74.3 | Primary biliary cirrhosis |
|  | K74.4 | Secondary biliary cirrhosis |
|  | K74.5 | Biliary cirrhosis, unspecified |
|  | K74.60 | Unspecified cirrhosis of liver |
|  | K74.69 | Other cirrhosis of liver |
| Viral Hepatitis | B18.0 | Chronic viral hepatitis B with delta-agent |
|  | B18.1 | Chronic viral hepatitis B without delta-agent |
|  | B18.2 | Chronic viral hepatitis C |
|  | B18.8 | Other chronic viral hepatitis |
|  | B18.9 | Chronic viral hepatitis, unspecified |
|  | B19.0 | Unspecified viral hepatitis with hepatic coma |
|  | B19.1 | Unspecified viral hepatitis B |
|  | B19.10 | Unspecified viral hepatitis B without hepatic coma |
|  | B19.11 | Unspecified viral hepatitis B with hepatic coma |
|  | B19.2 | Unspecified viral hepatitis C |
|  | B19.20 | Unspecified viral hepatitis C without hepatic coma |
|  | B19.21 | Unspecified viral hepatitis C with hepatic coma |
|  | B19.9 | Unspecified viral hepatitis without hepatic coma |
| Alcoholic Liver Disease | K70.0 | Alcoholic fatty liver |
|  | K70.1 | Alcoholic hepatitis |
|  | K70.10 | Alcoholic hepatitis without ascites |
|  | K70.11 | Alcoholic hepatitis with ascites |
|  | K70.2 | Alcoholic fibrosis and sclerosis of liver |
|  | K70.3 | Alcoholic cirrhosis |
|  | K70.30 | Alcoholic cirrhosis without ascites |
|  | K70.31 | Alcoholic cirrhosis with ascites |
|  | K70.4 | Alcoholic hepatic failure |
|  | K70.40 | Alcoholic hepatic failure without coma |
|  | K70.41 | Alcoholic hepatic failure with coma |
|  | K70.9 | Alcoholic liver disease, unspecified |
| Autoimmune & Other Hepatitis | K75.4 | Autoimmune hepatitis / lupoid NEC |
|  | K75.9 | Hepatitis, unspecified |
| Other Secondary Causes | E83.110 | Hereditary hemochromatosis / primary pigmentary cirrhosis |
|  | E83.119 | Other hemochromatosis |
|  | P78.81 | Congenital hepatic fibrosis |

This table lists the diagnostic codes used to identify cases of septic shock (inclusion), MASLD (exposure variable), and to exclude patients with cirrhosis/advanced fibrosis, viral hepatitis, alcoholic liver disease, autoimmune or other hepatitis, and other secondary causes.

**Supplementary Table S2: Sensitivity Analysis with Narrower Propensity Score Matching Caliper (0.1)**

| **Outcome** | **aOR** | **Lower CL** | **Upper CL** | **P value** |
| --- | --- | --- | --- | --- |
| Cardiac Arrest | 0.61 | 0.53 | 0.69 | <.0001 |
| AKI | 1.16 | 1.08 | 1.25 | <.0001 |
| Pulmonary Emboli | 0.49 | 0.43 | 0.62 | <.0001 |
| ARDS | 0.84 | 0.78 | 0.91 | <.0001 |
| Transfusion | 0.91 | 0.83 | 0.97 | 0.0051 |
| Death | 1.36 | 1.27 | 1.47 | <.0001 |

Adjusted odds ratios are presented with 95% confidence limits. This sensitivity analysis was performed using propensity score matching with a narrower caliper width of 0.1 to assess the robustness of the primary findings. P-values were derived from multivariable logistic regression models adjusted for baseline demographic and clinical characteristics. A two-sided P value <0.05 was considered statistically significant. aOR, adjusted odds ratio; AKI, acute kidney injury; ARDS, acute respiratory distress syndrome; CL, confidence limits.

**Supplementary Table S3: Sensitivity Analysis Excluding Patients with Baseline Chronic Kidney Disease**

| **Outcome** | **aOR** | **Lower CL** | **Upper CL** | **P value** |
| --- | --- | --- | --- | --- |
| Cardiac Arrest | 0.62 | 0.554 | 0.705 | <.0001 |
| AKI | 1.07 | 1.005 | 1.143 | 0.0356 |
| Pulmonary Emboli | 0.52 | 0.43 | 0.62 | <.0001 |
| Intubation ARDS | 0.83 | 0.784 | 0.888 | <.0001 |
| Transfusion | 0.90 | 0.83 | 0.97 | 0.0051 |
| Death | 1.283 | 1.203 | 1.369 | <.0001 |

Adjusted odds ratios are presented with 95% confidence limits. This sensitivity analysis excluded patients with baseline chronic kidney disease to evaluate the robustness of associations independent of pre-existing renal dysfunction. P-values were derived from multivariable logistic regression models adjusted for baseline demographic and clinical characteristics. A two-sided P value <0.05 was considered statistically significant. aOR, adjusted odds ratio; AKI, acute kidney injury; ARDS, acute respiratory distress syndrome; CL, confidence limits.
